# A Narrative Review on Dietary and Lifestyle Contributors to Non-Communicable Diseases in Gulf Cooperation Council Countries

**DOI:** 10.3389/phrs.2026.1609172

**Published:** 2026-04-28

**Authors:** Aaesha Salem Alhebsi, Tareq M. Osaili, Dimitrios Papandreou, Falak Zeb, Maysm N. Mohamad, Makhayel Sultan Alkaabi, Lily Stojanovska, Ayesha S. Al Dhaheri, Leila Cheikh Ismail

**Affiliations:** 1 Department of Clinical Nutrition and Dietetics, College of Health Sciences, University of Sharjah, Sharjah, United Arab Emirates; 2 Department of Nutrition and Food Technology, Faculty of Agriculture, Jordan University of Science and Technology, Irbid, Jordan; 3 Research Institute for Medical and Health Sciences, University of Sharjah, Sharjah, United Arab Emirates; 4 Department of Nutrition and Health, College of Medicine and Health Sciences, United Arab Emirates University, Al Ain, United Arab Emirates; 5 Institute for Health and Sport, Victoria University, Melbourne, VIC, Australia; 6 Nuffield Department of Women’s and Reproductive Health, University of Oxford, Oxford, United Kingdom

**Keywords:** body mass index (BMI), gulf cooperation council (GCC), lifestyle risk factors, non-communicable diseases (NCDs), physical activity (PA)

## Abstract

**Objectives:**

Non-communicable diseases (NCDs) are significant public health issue in the Gulf Cooperation Council (GCC). This review identified and evaluated dietary and lifestyle risk factors associated with NCDs across GCC countries.

**Methods:**

Google Scholar, Scopus, Web of Science, and PubMed were systematically searched for studies published between January 2020 and December 2024. Methodological quality was assessed using the Critical Appraisal Skills Programme (CASP).

**Results:**

Twenty studies met the inclusion criteria. Obesity prevalence ranged from 38% to 47% across GCC countries and emerged as a significant risk factor, with notable socioeconomic association, especially among women. The most prevalent unhealthy eating practices included excessive consumption of fast, processed, and ultra-processed foods, energy-dense diets, high-carbohydrate intake, and insufficient fruit and vegetable consumption. Educational level and income were strongly associated with dietary behaviors and physical activity. Physical inactivity was also consistently identified as a key contributor to obesity and NCD risk.

**Conclusion:**

Poor diet and sedentary behavior are major contributors to NCDs in the GCC. Targeted interventions, public education, promotion of active lifestyles, and supportive policy reforms are needed for both adults and children.

## Introduction

Dietary- and lifestyle-related non-communicable diseases (NCDs) are chronic, non-infectious conditions associated with poor dietary habits, including obesity, type 2 diabetes (T2D), cardiovascular diseases, hypertension, dyslipidemia, certain cancers, and non-alcoholic fatty liver disease [1, 2]. Unlike infectious diseases, NCDs develop gradually and are often influenced by genetic, physiological, environmental, lifestyle and behavioral factors [3]. For that reason, poor nutrition and lifestyle practices play a pivotal role in the development and progression of major NCDs [4].

Transition of poor nutrition characterized by increased consumption of added sugar, salt, and fat, with greater intake of animal products and ultra-processed foods (UPFs), while reducing intake of vegetables, fruits, and whole grain food products, is associated with an increased prevalence of nutrition related NCDs [5]. In addition, decreasing physical activity levels increases the risk of NCDs. Obesity, a significant risk factor for numerous chronic conditions, since 1975 has tripled globally in 2016 with over 1.9 billion adults were classified as overweight. [6]. T2D affects more than 537 million adults worldwide and is projected to rise 46% by 2045 [7]. NCDs represent the leading cause of mortality worldwide, accounting for 74% of all global deaths, approximately 41 million deaths annually [8]. These chronic conditions have reached epidemic proportions, with 17 million individuals dying before the age of 70 [9]. Cardiovascular diseases account for most NCDs fatalities, totaling 17.9 million deaths each year [10]. Nutrition-related NCDs significantly increase healthcare expenditures for prevention and treatment. Similarly, it leads to substantial productivity losses [11, 12].

The GCC countries, including Bahrain, Kuwait, Oman, Qatar, Saudi Arabia, and the United Arab Emirates, have experienced socio-economic transformation over recent decades [13]. These nations have transitioned from predominantly traditional lifestyles to highly urbanized societies with some of the highest *per capita* incomes globally. However, this rapid development has brought significant public health challenges, particularly in nutrition-oriented NCDs [14, 15].

Chronic diseases progressively dominate the epidemiological landscape in GCC countries. Diabetes prevalence in these nations ranks among the highest in the world, with estimates between 8% and 22% of individuals are diagnosed with T2D [16]. Cardiovascular disease mortality rates show significant regional variation, with Oman exhibiting the highest rate at 486.3 deaths per 100,000, followed by Qatar (337.3/100,000) and Saudi Arabia (332.1/100,000), while Bahrain reports the lowest at 240.9 deaths per 100,000 [17]. Ischemic heart diseases and strokes are the leading causes of CVD-related deaths in the GCC, accounting for over 78% of the total deaths [18]. Cancer incidence also varies across the region, with breast cancer rates increasing over time among women in most GCC countries, particularly in Bahrain, Kuwait, and Qatar [19]. Rapid urbanization and economic growth in the GCC have accelerated a shift away from traditional diets typically based on whole grains, dates, legumes, and fish toward more westernized dietary patterns characterized by higher intake of refined grains, sugar-sweetened beverages, processed meats, and energy-dense convenience foods. This transition has resulted in increased consumption of foods high in fat, added sugars, and sodium, alongside insufficient intake of fiber-rich fruits, vegetables, and whole grains, thereby contributing to the rising burden of obesity and cardiometabolic disorders in the region. Concurrently, physical inactivity has increased dramatically, with lower prevalence among women in the GCC than in men [20]. Individuals are increasingly physically inactive due to a lack of time, social support, suitable facilities, cultural norms, and the region’s hot climate [21]. Tobacco use is an important contributor to dietary- and lifestyle-related NCDs and is associated with an estimated 16.3% of cancer cases in the region. Sleep disorders may also contribute to NCD risk. In particular, obstructive sleep apnea is strongly associated with obesity [22, 23].

Understanding the poor dietary and lifestyle factors driving NCD prevalence in GCC countries is crucial for developing targeted, culturally appropriate interventions. GCC countries present unique contextual factors that may contribute distinctively to NCD development, including climate conditions limiting outdoor activity, cultural dietary practices, rapid wealth accumulation, high dependence on foreign diets, and subsidized food policies. In addition, emerging factors such as social media influence on dietary habits and health behaviors present additional challenges and opportunities, with studies showing significant relationships between social media exposure and unhealthy eating behaviors, while simultaneously offering platforms for health promotion and nutrition education [24–27].

This study will identify, evaluate, and synthesize evidence on dietary and lifestyle exposure related to major NCDs across GCC countries. By establishing the strength of associations between specific dietary patterns, nutritional factors, and NCD outcomes in this region. This study will provide an essential evidence for policymakers, healthcare providers, and public health practitioners.

## Methods

This paper conducted a systematic review to explore the lifestyle-related risk factors causing NCDs in the GCC countries, adhering to the Preferred Reporting Items for Systematic Reviews (PRISMA) [28]. This systematic review was registered on PROSPERO under number CRD420251123613. https://www.crd.york.ac.uk/PROSPERO/view/CRD420251123613.

### Data Sources

Comprehensive searches were undertaken across electronic databases (PubMed (22 March 2025; number of records = 52), Scopus (28 March 2025; number of records = 38), Google Scholar (2 April 2025; number of records = 187)) to provide a thorough understanding of the research landscape. To capture the latest trends and evidence, the search scope was restricted to papers published between January 2020 and December 2024.

### Search Strategy

Keywords used in the search strategy included nutrition-oriented non-communicable diseases NCDs, risk factors, obesity, diabetes, cardiovascular disease, diet, lifestyle, physical activity and terms specific to the countries of the Gulf Cooperation Council, such as Saudi Arabia, Kuwait, Oman, Qatar, Bahrain, and the United Arab Emirates. These keywords were combined with Boolean operators (AND, OR) to formulate the search strings.

### Study Selection

The records retrieved from the database searches were screened in two phases: titles and abstracts screening, followed by a comprehensive review of the full texts against prespecified eligibility criteria. The main outcome of this review was to explore the dietary and lifestyle factors that contribute to the development of NCDs in the GCC region from childhood to adulthood.

### Eligibility Criteria

This review included peer-reviewed articles encompassing original research, including observational studies published in English investigating nutrition-oriented risk factors such as dietary habits, obesity, sedentary lifestyles, and other pertinent nutritional behaviors contributing to NCDs in the GCC countries. Studies that identified dietary and lifestyle factors affecting the course of NCDs in both male and females were included. This review excluded letters, editorials, conference abstracts, unpublished data, study protocols, reviews, meta-analyses, and opinion pieces. Studies were also excluded if they did not investigate lifestyle-oriented risk factors or address NCDs, were conducted outside the GCC countries or involving non-relevant populations, or were published before 2020. This date restriction ensured the evidence reflects the recent GCC policy context and post-2020 changes, including pandemic disruptions to diet and physical activity.

### Quality Assessment

The quality of the selected studies was evaluated using the Critical Appraisal Skills Programme (CASP) tool. The tool offers a systematic framework for assessing multiple dimensions of study quality, including the clarity of the research question, suitability of study design, sample size, risk of bias, and relevance of findings. Conflicts in assessment were reconciled by discussion and/or the intervention of a third review author. The quality assessment facilitated the evaluation of the research’s reliability and validity, prioritising higher-quality studies for inclusion in the final analysis.

### Data Extraction

Two independent authors extracted study characteristics, including the author, year of publication, study design, participants’ demographics, and nutrition-oriented risk factors such as dietary habits, obesity, and physical activity from the included studies using a standardized form. The extraction form was updated to capture age/age group, sex/gender, nationality/ethnicity (where reported), education, socioeconomic status (SES)/income, comorbidities/chronic conditions, exposure measurement tools, outcome definitions, and statistical methods/adjustments. Extracted variables were incorporated into Table 1 when available; where information was absent, “NR” (not reported) was recorded.

**TABLE 1:** Study characteristics, dietary and lifestyle factors associated with non-communicable diseases (Gulf Cooperation Council countries, 2020–2024).

References	Country	Objective	Study design and data Source	Sample characteristics	NCDs risk factors/Focus	Key findings	Recommendation
[29]	Saudi Arabia	To provide empirical evidence on socioeconomic and demographic correlates of NCD risk factors among adults in Saudi Arabia	Cross-sectional study using secondary data from the 2013 Saudi Health Interview Survey (SHIS), Probability proportional to size sampling	10,735 adults, aged 15+ years across all regions of Saudi Arabia	Tobacco use, low fruit/vegetable consumption, low physical activity	- High prevalence of risk factors: tobacco use (12.1%), low fruit/vegetable consumption (87%), low physical activity (94.9%), overweight/obesity (65.1%), hypertension (37.5%) - Significant correlates for overweight/obesity and hypertension include gender, employment type, income, and education - Significant lifestyle associations for tobacco use, diet, and activity	Calls for reducing life-damaging behaviours and promoting healthy lifestyles (e.g., physical activity, diet) to target various socioeconomic groups and reduce chronic NCD prevalence
[30]	Saudi Arabia	To examine the socioeconomic determinants and inequalities in NCD prevalence in Saudi Arabia	Cross-sectional study using 2018 data from the Saudi Family Health Survey	11,527 respondents, aged 18 and above, 45.71% female vs. 45.29% male	Socioeconomic and regional inequalities	- The prevalence of NCDs is 32.15%, with higher prevalence among women and elderly (60+ years) - Lower among individuals with higher education levels, and more prevalent among less educated and lower-income women	Recommends targeted interventions to combat NCD prevalence and reduce socioeconomic inequalities, with a focus on women and less-educated/lower-income groups to achieve health equity
[31]	Saudi Arabia	To examine the association between the number of NCDs and physical activity levels in older adults	Cross-sectional study. Data collected using self-reported PA Scale for the Elderly (PASE) and NCD data	94 adults aged ≥60 (62 men and 32 women) with a mean age of 67.29 years	Physical activity	- Significant negative association between number of NCDs and physical activity, even after adjustment for confounders	Recommends physical activity promotion among older adults to prevent or reduce NCDs
[32]	Kuwait	To identify dietary patterns in Kuwaiti adults and examine associations with CVD risk factors	Cross-sectional study using data from National Nutrition Survey of Kuwait	555 Kuwaiti adults aged ≥20 years	Obesity, abdominal obesity, high BP, dyslipidemia, diabetes, metabolic syndrome	- Identified three dietary patterns: vegetable-rich, fast food, refined grains/poultry - Fast-food pattern positively associated with BMI, waist circumference and BP - Refined grains/poultry pattern associated with elevated glucose	Needed further prospective studies on dietary patterns and CVD among at-risk populations. Calls for dietary interventions targeting at-risk groups
[33]	Kuwait	To evaluate the prevalence and association of overweight, obesity, and central obesity with socio-demographic factors in Kuwait	Cross-sectional survey conducted as part of Kuwait diabetes Epidemiology Program. Used WHO STEPwise approach	4,901 adults aged 18–82 years. Mostly non-Kuwaiti nationals (76%)	Overweight, obesity, central obesity	- Overweight prevalence: 40.6%, obesity: 42.1%, central obesity: 73.7% - Overweight greater in men, obesity/central obesity higher in women - Higher educational attainment, physical activity linked with lower odds of obesity	Highlights need for early prevention through mandatory physical education and recreational spaces. Recommends policies promoting physical activity and lifestyle changes for young adults
[34]	Kuwait	To estimate the number and proportion of cardiometabolic deaths attributable to suboptimal dietary intake among Kuwaiti adults	Cross-sectional study incorporating dietary intake data from national nutrition survey and WHO data	Kuwaiti adults aged 25+ years, with data from the 2009 national nutrition survey	Low intake of nuts/seeds, high sodium, low fruit/vegetable intake	- 1,308 cardiometabolic deaths were attributed to poor diet (64.7% of all cardiometabolic deaths in Kuwait) - Low intake of nuts/seeds had the highest association (18.8%), followed by high sodium intake, and low fruit and vegetable intake - Men and young adults experienced the largest proportion of diet-attributable deaths	Highlights the need to improve dietary habits in Kuwait, with targeted interventions for young adults and men to reduce diet-associated cardiometabolic deaths
[35]	Kuwait	To analyse dietary habits, meal timing, and meal frequency among Kuwaiti adults	Analysis of Kuwait National Nutrition Surveillance System data	757 Kuwaiti adults, aged 20 and above, 45.05% male vs. 54.95% female	Meal timing, meal frequency, skipping meals, late-night eating	- 54% of Kuwaiti adults eat after 10 p.m.; 29% skip breakfast, and 9.8% skip dinner - Women skip breakfast more often and engage in more extended night fasting than men - Married adults skip breakfast and dinner less than unmarried adults	Emphasizes the need to further investigate how meal timing and frequency influence the prevalence of NCDs among Kuwaiti adults
[36]	Bahrain	To present and analyse Bahrain’s food-based dietary guidelines (FBDG) and their holistic approach to health	Descriptive study with situation analysis of diet-related diseases and food consumption patterns in Bahrain	Healthy adult Bahraini population,	Dietary habits, and vitamin D deficiency	- Low fruit and vegetable intake and excessive consumption of processed meat and sugary drinks noted, - Bahrain FBDG comprised 11 themes covering body (e.g., diet, physical activity), mind (e.g., mental health, mindful eating), society (e.g., cultural heritage), and environment (e.g., food waste)	Promotes a holistic approach to dietary recommendations by addressing physical health, mental wellbeing, social, and environmental aspects of dietary habits
[37]	Bahrain	To assess Type 2 diabetes (T2D) awareness among the non-medical Bahraini population	Cross-sectional study using an electronic survey (Google Forms)	835 participants aged 15 and above, non-healthcare workers	Type 2 diabetes awareness	Average overall T2D awareness was 70.6%, Higher awareness in diabetic population (76.7%) vs. non-diabetic (72.5%)	Need for educational programs and strategic use of social media to improve T2D awareness in Bahrain and reduce its prevalence
[38]	Oman	To investigate the relationship between Nutrition Quality of Life (NQOL) and affective functioning in Omani patients with type 2 diabetes	Cross-sectional study with face-to-face interviews	149 Omani patients with Type 2 diabetes from 7 Primary Health Centers	Effective functioning, nutrition, quality of life	- Poor glycemic control (71.1%) - Negative correlation between NQOL and anxiety/depression (r = −0.590, P < 0.0001)	Significant interventions to improve quality of life and mental health in diabetes patients
[39]	Oman	To estimate the prevalence of micronutrient deficiencies, anaemia, genetic blood disorders, and malnutrition in women and children	National cross-sectional survey	Women of reproductive age (approximately 4,100) and children aged 0–59 months (approximately 2,500–3,000)	Micronutrient deficiencies, anaemia, over- and undernutrition	- 23.8% of children were anaemic - 59.2% of women were overweight or obese - Sickle cell and β-thalassaemia traits in 5.3% of children	Anaemia, iron deficiency, and overweight/obesity in women are significant nutritional problems in Oman. Calls for targeted nutrition programs to address these issues
[40]	Oman	To assess the impact of socio-demographic factors on obesity in Omani women aged 30–49 years	Cross-sectional study with questionnaires	398 Omani women aged 30–49 years with BMI >30	Socio-demographic factors	- 47% were class I obese (BMI 30–35) - Obesity is associated with marital status, income, and family structure	- Health awareness programs that promoting a healthy lifestyle
[41]	Oman	To assess the consumption, willingness to consume, availability, and knowledge of whole grain food products across Oman	Cross-sectional study with a self-administered survey among a convenience sample	1891 adults across nine Governorates of Oman	Consumption, knowledge, and availability of whole grain products	- 99% consume rice and bread at least 4 days/week - Less than 5% consume brown rice - 20%–90% consume whole grain bread, and 40% are unsure which rice or bread is healthier	Urges education on the nutritional benefits of whole grain products and improvement in availability across Oman
[42]	United Arab Emirates	To determine the prevalence of obesity, its associated factors, and complications among adults at Hatta Hospital, UAE.	Cross-sectional study at Hatta Hospital, between January–August 2022	600 Emirati adults aged 18–55 years	Unhealthy diet and physical inactivity	- 38.5% obese, 33.7% overweight, 72.2% of the adult population in Hatta has high BMI. - Obesity is linked to lower education, unhealthy diet, and physical inactivity	Highlights the need for interventions focusing on education and lifestyle changes
[43]	United Arab Emirates	To study the prevalence of overweight and obesity and determine the associated risk factors among adults in Dubai	Cross-sectional survey with multistage, stratified random sampling	2142 adults aged 18+ years in Dubai	Overweight and sociodemographic risk factors	- 17.8% prevalence of obesity, higher in women (21.6%) and UAE nationals (39.6%), 39.8% overweight - Obesity is associated with age, sex, nationality, occupation, and hypertension	Calls for comprehensive initiatives to control obesity, particularly among high-risk groups such as UAE nationals and service workers
[44]	Qatar	To assess the prevalence of overweight and obesity among school-going students in Qatar	Cross-sectional study using data from 332 school campuses (2016–17)	186,986 students, 5–19 years, Male n = 90,833 vs. female n = 96,153	Overweight and obesity in students	- 42.3% of students were overweight or obese - Highest prevalence in Qatari male students (47.1%), followed by Qatari females (43.8%), Qatari males and non-Qatari males were predominantly more obese, while females were more overweight	Need public health interventions to combat rising obesity rates among students
[45]	United Arab Emirates	To examine the anthropometric status, food consumption patterns, and nutrient intake of children in the UAE	Cross-sectional survey using 24-h recall and anthropometric data from 690 children (4–12.9 years)	690 children from 3 Emirates, 4–13 years, 47.8% boys vs. 52.2% girls	Nutrient intake, overweight, stunting, and obesity	- 4% of children were stunted, 8% were wasted, and 28% were overweight/obese - High intake of free sugars and saturated fats, while low intakes of essential fatty acids and vitamins D, A, calcium	Significant deficiencies in children’s diets calls for targeted nutritional policies to improve adherence to dietary recommendations, especially for fruits, vegetables, and dairy
[46]	United Arab Emirates	To investigate vitamin D deficiency and its risk factors among female migrants in the UAE	Cross-sectional study with among migrants in Al Ain, UAE	550 females, 18 years and above	Vitamin D deficiency and associated risk factors	- 67% of participants had vitamin D deficiency. Higher deficiency in Arabs (87%) and South Asians (83%), lower in Filipinas (15%) - Risk factors included low physical activity, obesity, and long residence in the UAE	Females should be educate for increased sun exposure, physical activity, and supplementation to address this health issue
[47]	UAE (Sharjah)	To assess Mediterranean Diet adherence among adults and identify influential predictors	Cross-sectional study with a self-reported, web-based questionnaire	1314 participants (age 25–52 years), 822 females (62.6%) and 492 males (37.4%)	Mediterranean Diet adherence and lifestyle factors	- Moderate adherence to the MD (score 5.9 ± 1.9). Low adherence to fish, fruits, and legumes - Physical activity, nutrition information from dietitians, and social media positively associated with higher adherence	Significant predictors for higher MD adherence: being married, physically active, non-smoker, and getting nutrition information from dietitians and social media Public health and nutrition specialists should tailor approaches to promote MD adherence
[48]	Qatar	To assess the impact of COVID-19-related school closures on children’s and adolescents’ diet and physical activity	Analytical cross-sectional study using national electronic health records and telephone interviews with parents	1546 participants, 845 (54.7%) aged 8–11 years, and the remaing 12–15 years	Impact of COVID-19 school closures on diet and physical activity	- Decreased vegetable intake and increased consumption of soft drinks, fried foods, fast food, and sweets - Reduced physical activity during school closure - Higher parental education, maternal employment, and family history of obesity are linked to adverse lifestyle changes	Emphasises the need for interventions to promote healthy behaviours during disruptions and long-term strategies to address NCD risks

### Data Analysis

The data was analyzed thematically to determine repeating themes or patterns, such as the prevalence and impact of lifestyle-related NCDs in the GCC region [49]. The analysis identified the primary lifestyle risk variables, mainly in terms of unhealthy eating and sedentary lifestyles. Using the prevalence of the risk variables and the association with NCD outcomes, thematic categories were developed. A qualitative synthesis method was employed to encapsulate the principal findings, encompassing prevalence rates of NCDs and the link lifestyle-associated risk variables. The results were structured to emphasise trends, deficiencies, and implications for public health interventions in the GCC region.

## Results

### Study Selection

**FIGURE 1:**
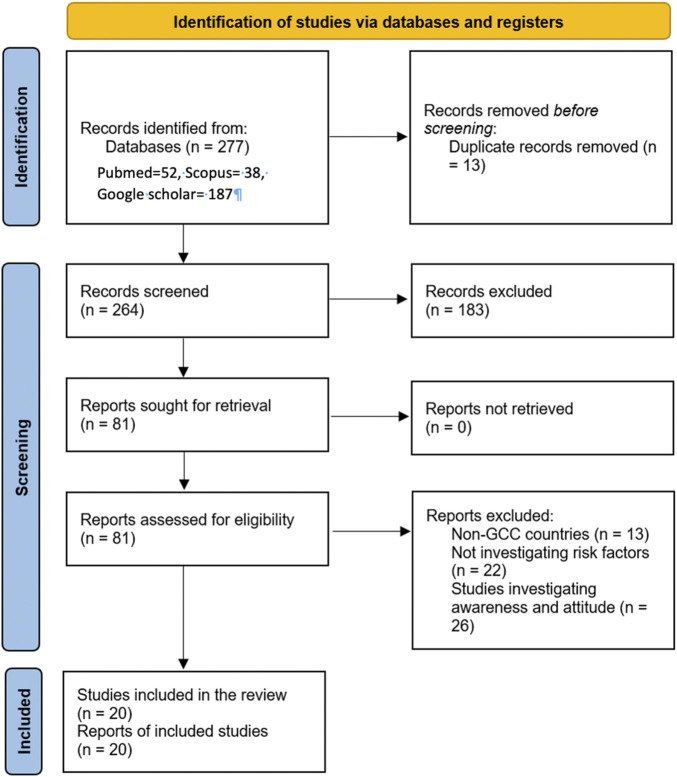
Preferred Reporting Items for Systematic Reviews and Meta-Analyses flow diagram (A Narrative Review on Dietary and Lifestyle Contributors to Non-Communicable Diseases in Gulf Cooperation Council Countries, Gulf Cooperation Council countries, 2020–2024).

The total number of records identified in the databases was 277. Thirteen duplicates and 99 records published before 2020 were removed. One hundred sixty-five records were screened, after which 84 were excluded following title and abstract screening. Eighty-one full-text articles were retrieved and meticulously screened. Twenty-two studies did not focus on risk factors, 13 studies encompassing additional nations alongside the GCC countries, and 26 studies that concentrated solely on awareness and attitude were excluded. Twenty research articles that met the eligibility criteria were included in this analysis (Figure 1).

### Study Characteristics

This review included 19 cross-sectional studies, and one descriptive study. *The majority of included studies employed cross-sectional designs (n = 18, 90%), reflecting the predominance of observational epidemiological evidence in the region.* These studies were conducted in different GCC countries and focused on various risk factors for NCDS, dietary habits, and lifestyle factors. These studies employed secondary data, national surveys, and self-reported questionnaires. Sample sizes varied from 100 to more than 180,000 participants, with an approximate mean sample size of ∼11,800 participants, reflecting the inclusion of several large national surveys. Most studies included large population samples, which helped reduce the role of random error. Among the included studies, most were conducted among adult populations (n = 12, 60%), while fewer studies focused on children or adolescents (n = 3, 15%), patients with specific conditions such as diabetes (n = 2, 10%), or migrant populations (n = 1, 5%). Regarding health outcomes, obesity and overweight were the most frequently investigated conditions (n = 8, 40%), followed by cardiometabolic risk factors including hypertension and cardiovascular disease (n = 4, 20%), while type 2 diabetes was specifically addressed in two studies (10%). The studies examined significant risk factors associated with NCDs, including obesity, hypertension, tobacco consumption, lack of physical activity, and dietary habits. In addition, the connections between these factors and various socio-demographic characteristics, such as age, gender, education, and income levels, were analyzed (Tables 1, 2).

**TABLE 2:** Outcomes and key results reported in the included studies (Gulf Cooperation Council countries, 2020–2024).

Reference	Outcomes	Analysis model	Key results (effect sizes + p-values)
[29]	Multiple NCD risk factors (e.g., smoking; physical inactivity; overweight/obesity; hypertension; diabetes; hypercholesterolemia; diet indicators such as low fruit/vegetable intake)	Descriptive statistics; multivariable logistic regression with adjusted odds ratios (AORs) and 95% CIs (table uses significance markers rather than listing exact p-values for AORs)	Adjusted associations (AOR, 95% CI): female vs. male—overweight/obesity 1.20 (1.07–1.35), hypertension 1.54 (1.34–1.77), diabetes 1.62 (1.24–2.11), physical inactivity 2.19 (1.90–2.52). Strong age gradients were reported (e.g., age 55–64 vs. 15–24: overweight/obesity 7.69 (6.20–9.54), hypertension 10.86 (8.25–14.30), diabetes 34.99 (18.92–64.71)). Exact p-values for AORs were not shown in the displayed regression table; significance was indicated via table notation
[30]	Presence of NCD and socioeconomic inequality (concentration index and decomposition), plus correlates (age, sex, education, income, etc.)	Descriptive statistics; logistic regression (ORs with p-level notation); inequality metrics (concentration index) and decomposition	Illustrative correlates from reported regression (ORs; p shown as thresholds via significance notation): older age categories were associated with higher odds (e.g., the oldest group had markedly higher OR vs. youngest), men had higher odds than women, and higher income/education were associated with lower odds in the presented models. Exact numeric p-values were not systematically reported (stars/thresholds used)
[31]	Number of NCDs and age as predictors	Linear regression with multiple models (unadjusted; adjusted for sociodemographic factors; further adjusted for smoking and BMI)	NCD count → lower PA: fully adjusted model (Model 3) β = −8.08 (SE 3.64), p = 0.02 (each additional NCD associated with ∼8-point lower PASE score). Age was also inversely associated with PA in adjusted models (Model 3: β = −1.56, SE 0.50, p = 0.00 as reported)
[32]	Outcomes include BMI, waist circumference, blood pressure, plasma glucose, metabolic syndrome (and other cardiometabolic risk factors)	Principal component analysis; survey-weighted multivariable linear and logistic regression (as described in the article preview)	Reported linear-regression associations (β, 95% CI) from the preview: fast-food pattern associated with higher BMI β = 0.94 (0.08–1.79), waist circumference β = 2.05 cm (0.20–3.90), diastolic BP β = 1.62 mmHg (0.47–2.77). Refined grains/poultry pattern associated with higher plasma glucose β = 1.02 mg/dL (1.002–1.04). Approximate p-values derived from these 95% CIs (Wald approximation): BMI p ≈ 0.031, waist circumference p ≈ 0.030, diastolic BP p ≈ 0.0058, plasma glucose p < 0.001 (approx.). The preview states higher odds of metabolic syndrome in highest vs. lowest tertile of certain patterns, but numerical ORs/p-values for metabolic syndrome were not provided in the accessible preview text
[33]	Overweight, obesity, and central obesity	Weighted prevalence estimation; univariate and multivariable logistic regression with OR/AOR, 95% CI, and p-values	Prevalence (weighted): overweight 40.6%, obesity 42.1%, central obesity 73.7%. Key adjusted associations (examples): overweight—female vs. male AOR 0.79 (0.64–0.99), p = 0.04. Obesity—female vs. male AOR 1.54 (1.19–1.99), p < 0.001; higher income >1500 vs. ≤500 AOR 2.17 (1.41–3.33), p < 0.001; current smoker vs. non-smoker AOR 2.54 (1.02–6.36), p = 0.05; WHO-defined elevated BP AOR 2.34 (1.59–3.44), p < 0.001. Central obesity—female vs. male AOR 7.39 (5.21–10.49), p < 0.001; non-Kuwaiti vs. Kuwaiti AOR 0.58 (0.38–0.87), p = 0.01; higher income >1500 vs. ≤500 AOR 2.25 (1.40–3.61), p < 0.001
[34]	Estimated number and proportion of cardiometabolic deaths	Comparative Risk Assessment (CRA) modeling, Monte Carlo simulation to generate 95% uncertainty intervals	Estimated 1,308 cardiometabolic deaths attributable to suboptimal diet (95% UI 1,228–1,485), representing 64.7% (95% UI 60.7%–73.4%) of cardiometabolic deaths in Kuwait in 2009. Leading dietary contributors by estimated deaths included low nuts/seeds (n = 380, 18.8%), high sodium (n = 256, 12.6%), low fruits (n = 250, 12.4%), low vegetables (n = 236, 11.7%), low whole grains (n = 201, 9.9%), high SSBs (n = 201, 9.9%). This is a modeling study reporting uncertainty intervals, not regression coefficients/p-values
[35]	Meal timing (late-night eating), meal frequency, breakfast/dinner skipping, night fasting duration (derived from 24h dietary recall)	Descriptive secondary analysis using surveillance data	Key prevalence estimates: ∼54% ate after 10 p.m.; 29% skipped breakfast; 9.8% skipped dinner; mean 4.4 meals/day. Sex differences: women skipped breakfast more and had longer night fasting than men (p < 0.001); late-night eating differed by sex (p = 0.0373). Marital status differences: unmarried participants skipped breakfast and dinner more than married participants (p < 0.001 as reported)
[36]	Diet-related disease	Situation analysis + synthesis using mixed-method content and thematic analyses	Produced 11 context-specific guideline themes aligned with a “biopsycho-ecological” framework. Reports descriptive situational indicators such as high prevalence of inadequate fruit/vegetable intake (e.g., 85% consuming < recommended) and other dietary habit burdens, as part of the situation analysis (drawn from available data sources). No study-level β/OR results are applicable because this is not a regression-based association study
[37]	Type 2 diabetes (overall and by knowledge domains)	Descriptive analysis in spreadsheet software; reporting means, SDs, percentages	Among 613 non-medical participants, overall awareness was 70.6% (CI ± 6.214; SD ± 8.80). Awareness was higher among participants with diabetes (76.7%) vs. non-diabetic (72.5%). Domain scores included general knowledge ∼54.5%, risk factors ∼75.5%, symptoms ∼77.6%, complications ∼61.8%, treatment/monitoring/prevention domain summaries as reported. No β/OR associations reported
[38]	Obesity and glycemic control	Pearson correlation and χ^2^ tests; effect size reporting via Cramér’s V; stratified/controlled association analyses	Key associations: NQOL inversely correlated with HADS total (r = −0.590, P = 0.000 as reported), anxiety (r = −0.597, P = 0.000), and depression (r = −0.435, P = 0.000). χ^2^ association between NQOL and HADS: χ^2^(2) = 38.21, P < 0.01, Cramér’s V = 0.51; additional controlled associations reported with Cramér’s V ∼0.43–0.55 at P < 0.01 (controlling for HbA1c/BMI/waist/HMNT)
[39]	Prevalence of anemia, iron/vitamin deficiencies, genetic blood disorders; underweight/overweight/obesity	Prevalence estimation; Regression coefficients	Key prevalence (as reported): children 0–59 months—wasting 9.3%, stunting 11.4%, overweight/obese 4.2%; anemia 23.8%; iron deficiency 10.2%, vitamin A deficiency 9.5%, vitamin D deficiency 10.6%. Women 15–49—underweight 9.1%, overweight/obese 59.2%; anemia 27.8%; iron deficiency 24.8%, folate deficiency 11.6%, vitamin B12 deficiency 8.9%, vitamin D deficiency 16.2%. Genetic traits: sickle cell and β-thalassemia traits noted in both children and women at the reported prevalences
[40]	Obesity class (I/II/III) among obese women; waist–hip ratio	Descriptive statistics; chi-square tests stated with p < 0.05 threshold	Among n = 398 obese women (BMI >30), obesity classes: class I 47% (n = 187), class II 32% (n = 126), class III 21% (n = 85). Central obesity marker: report states ∼70% had WHR >0.85. Table-based distributions are provided by education, marital status, occupation, income, and region, but row-wise p-values/χ^2^ statistics are not displayed in the table image itself (limiting quantification of “association strength” beyond descriptive differences)
[41]	Whole-grain consumption frequency; knowledge of healthier grain choices; perceived market availability; indices (knowledge/availability/willingness)	Survey across nine governorates; descriptive statistics; χ^2^ tests to assess effects of governorate, sex, income, and education on outcomes (α = 0.05); index construction for availability/knowledge/willingness	Sample/process: 2,773 questionnaires distributed; 1,891 returned (68%). Descriptive findings: 99% reported consuming rice and bread ≥4 days/week; <5% consumed brown rice; 40% did not know which rice/bread type is healthier; only 20%–30% reported brown rice/whole wheat bread availability “all the time.” Indices ranged by governorate: availability 0.36–0.87, knowledge 0.35–0.64, willingness 0.51–0.57. Specific χ^2^ statistics/p-values for subgroup differences were not extracted from the accessible text view
[42]	BMI andobesity; NCD complications by BMI group	Descriptive stats (median/IQR); normality testing stated; chi-square comparisons across BMI categories; ordinal logistic regression and multivariable model	Prevalence: underweight 4.3%, normal 23.5%, overweight 33.7%, obese 38.5% (BMI categories). Multivariable (Table 3) examples (OR, 95% CI, p): illiterate vs. university OR 5.4 (1.9–15.3), p = 0.002; middle school vs. university OR 77.2 (3.9–383.4), p = 0.002; high school vs. university OR 5.6 (1.4–6.1), p = 0.005; college vs. university OR 2.7 (1.3–5.5), p = 0.002. Single vs. married OR 0.6 (0.3–0.9), p = 0.011. Healthy vs. unhealthy diet OR 0.4 (0.3–0.5), p < 0.001
[43]	Population prevalence of overweight and obesity; and risk factors	Multivariate logistic regression	Snippet-level result available: overweight prevalence reported as 39.8%; multivariate logistic regression stated to assess associations. Detailed adjusted coefficients/ORs and p-values could not be extracted here due to access restrictions on the full text in this environment
[44]	BMI-for-age z-score	z-test; survival, or longitudinal models	Overall overweight/obesity prevalence was 42.3%. Boys had higher odds than girls (OR 1.21, 95% CI 1.19–1.24), and Qataris had higher odds than non-Qataris (OR 1.23, 95% CI 1.21–1.26). Obesity was higher in boys (OR 1.50, 95% CI 1.46–1.53) and in Qataris (OR 1.47, 95% CI 1.43–1.51). Students aged 10–14 years had the highest odds of overweight/obesity vs. ages 5–9 (OR 1.76, 95% CI 1.72–1.80; p < 0.001). Non-Qatari females were less likely to be overweight than Qatari females (OR 0.79, 95% CI 0.77–0.81; p < 0.001). Differences by school level, municipality, and school type were significant (p < 0.0001)
[45]	anthropometric/nutritional status indicators	Cross-sectional survey reporting “usual intake” tables and p-values for age-group differences in dietary contribution from food groups;	Mean food-group intake by age group with p-values for age-group differences, and usual macro/micronutrient intakes and % compliance with DRIs. Specific numeric results were not captured in the current tool outputs, so effect sizes/p-values are not reproduced here
[46]	Vitamin D deficiency (25(OH)D ≤20 ng/mL) among female migrants; predictors of obesity	chi-square and t-test; ordered logistic regression and binary logistic regression; multivariable logistic regression for correlates (AOR with 95% CI)	After adjustment, significant correlates included: low physical activity AOR 4.59 (95% CI 1.98–10.63); >5 years residence AOR 4.65 (95% CI 1.31–16.53); obesity AOR 3.56 (95% CI 1.04–12.20). Exact p-values for these AORs were not printed in the narrative sentence; significance is implied by inclusion in “independently associated” correlates and/or table formatting. Approximate p-values from CI (Wald): low physical activity p ≈ 0.00038, >5 years residence p ≈ 0.017, obesity p ≈ 0.043
[47]	Mediterranean diet adherence (MD score) as continuous outcome; sociodemographic, health, and information-source predictors	Multiple linear regression (β with 95% CI; p < 0.05). Includes Model 1 and Model 2 (Model 2 described as adjusted for age and sex in table note)	Non-Mediterranean nationality β −0.266 (95% CI −0.472 to −0.059), p = 0.012; marital status β 0.277 (0.038–0.516), p = 0.023; smoking β −0.406 (−0.698 to −0.113), p = 0.007; type of physical activity β 0.747 (0.509–0.984), p < 0.001; dietitian as a diet-regimen source β 0.602 (0.269–0.934), p < 0.001; social media as a diet-regimen source β 0.538 (0.252–0.823), p < 0.001
[48]	Changes in dietary behaviors and physical activity during school closures, and sociodemographic predictors of adverse changes (e.g., increased soft drinks, fried food, fast/junk food, sweets; reduced physical activity)	Descriptive statistics; univariate tests (χ^2^/Fisher, t-test/Mann–Whitney as appropriate); multivariable logistic regression with AOR and 95% CI; Hosmer–Lemeshow goodness-of-fit; p < 0.05 threshold	Example outcomes and predictors (AOR, 95% CI, p): increased soft drinks/sweetened beverages—father age ≥55 vs. ≤35 AOR 3.42 (1.24–9.47), p = 0.018; females less likely than males AOR 0.67 (0.50–0.90), p = 0.007; mother with no formal education vs. college+ AOR 0.26 (0.07–0.93), p = 0.038. Increased junk/fast food—father 45–54 AOR 2.67 (1.02–6.96), p = 0.045; employed mother AOR 1.70 (1.28–2.26), p < 0.001; family history of obesity/overweight AOR 1.58 (1.21–2.07), p = 0.001. Increased sweets—younger developmental stage AOR 1.74 (1.37–2.21), p < 0.001; employed mother AOR 1.55 (1.21–2.00), p = 0.001. Reported change tests also included, e.g., fruit intake changes p = 0.459 (NS), vegetable intake decreased p < 0.001, soft drinks increased p = 0.021

### Quality Assessment

All 20 studies included in the appraisal demonstrated high methodological quality and relevance to the research objectives. Each study addressed a clearly focused issue and employed an appropriate methodology to answer the research question. The recruitment processes were acceptable across all studies, and measures were applied accurately to reduce potential bias. Regarding recruitment strategies, probability-based sampling methods were used in 5 studies (25%), including national surveys and stratified sampling approaches [29, 37, 40, 50, 51]. Convenience sampling was employed in 7 studies (35%) [30, 32, 34, 39, 43, 52, 53], while cluster or institutional sampling (e.g., schools, hospitals, or health centers) was used in 4 studies (20%) [32, 33, 36, 38]. The remaining 4 studies (20%) relied on secondary national datasets or surveillance systems [29, 35, 42, 45]. Data collection methods were well-aligned with the research aims, and all studies had an adequate sample size to minimise the role of chance. Data analysis was conducted with sufficient rigor, and every study provided a clear statement of findings. In terms of analytical rigor, multivariable or adjusted regression models were used in 9 studies (45%) to control for potential confounders [29, 30, 33, 36, 40, 42, 50, 51, 53]. Descriptive and prevalence-based analyses were reported in 8 studies (40%) [32, 37–39, 43–45, 52], while comparative risk modelling or advanced epidemiological modelling approaches were used in 1 study (5%) [35]. Additionally, subgroup analyses based on demographic factors such as sex, age, nationality, or socioeconomic status were conducted in 6 studies (30%), further strengthening the interpretability of the findings. Moreover, the results from all studies were deemed applicable to the local population and were considered highly valuable in informing practice and policy (Table 3). Overall, the appraisal revealed a consistent pattern of methodological robustness and practical significance across the reviewed literature.

**TABLE 3:** Methodological quality assessment of the included studies using the Critical Appraisal Skills Programme tool (Gulf Cooperation Council countries, 2020–2024).

Author, year	Did the study address a focused issue?	Did the authors use an appropriate method to answer their question?	Were the subjects recruited in an acceptable way?	Were the measures accurately measured to reduce bias?	Were the data collected in a way that addressed the research issue?	Did the study have enough participants to minimise the play of chance?	Was the data analysis sufficiently rigorous?	Is there a clear statement of findings?	Can the results be applied to the local population?	How valuable is the research?
[30]	Yes	Yes	Yes	Yes	Yes	Yes	Yes	Yes	Yes	Highly
[29]	Yes	Yes	Yes	Yes	Yes	Yes	Yes	Yes	Yes	Highly
[31]	Yes	Yes	Yes	Yes	Yes	Yes	Yes	Yes	Yes	Highly
[32]	Yes	Yes	Yes	Yes	Yes	Yes	Yes	Yes	Yes	Highly
[33]	Yes	Yes	Yes	Yes	Yes	Yes	Yes	Yes	Yes	Highly
[34]	Yes	Yes	Yes	Yes	Yes	Yes	Yes	Yes	Yes	Highly
[35]	Yes	Yes	Yes	Yes	Yes	Yes	Yes	Yes	Yes	Highly
[36]	Yes	Yes	Yes	Yes	Yes	Yes	Yes	Yes	Yes	Highly
[37]	Yes	Yes	Yes	Yes	Yes	Yes	Yes	Yes	Yes	Highly
[38]	Yes	Yes	Yes	Yes	Yes	Yes	Yes	Yes	Yes	Highly
[39]	Yes	Yes	Yes	Yes	Yes	Yes	Yes	Yes	Yes	Highly
[40]	Yes	Yes	Yes	Yes	Yes	Yes	Yes	Yes	Yes	Highly
[41]	Yes	Yes	Yes	Yes	Yes	Yes	Yes	Yes	Yes	Highly
[42]	Yes	Yes	Yes	Yes	Yes	Yes	Yes	Yes	Yes	Highly
[43]	Yes	Yes	Yes	Yes	Yes	Yes	Yes	Yes	Yes	Highly
[44]	Yes	Yes	Yes	Yes	Yes	Yes	Yes	Yes	Yes	Highly
[45]	Yes	Yes	Yes	Yes	Yes	Yes	Yes	Yes	Yes	Highly
[46]	Yes	Yes	Yes	Yes	Yes	Yes	Yes	Yes	Yes	Highly
[47]	Yes	Yes	Yes	Yes	Yes	Yes	Yes	Yes	Yes	Highly
[48]	Yes	Yes	Yes	Yes	Yes	Yes	Yes	Yes	Yes	Highly

### Obesity and Associated Socioeconomic Factors

Many included studies underscore the significant prevalence of obesity and associated risk factors within the GCC nations. There was a considerable prevalence of various risk factors, including tobacco use (12.1%), inadequate fruit and vegetable consumption (87%), insufficient physical activity (94.9%), overweight and obesity (65.1%), and hypertension (37.5%) [29]. In addition, there were socioeconomic associations with overweight/obesity and hypertension, encompassing variables such as gender, employment type, income, and educational attainment. Moreover, there was a general prevalence of non-communicable diseases (NCDs) in Saudi Arabia (32.15%), with elevated rates observed among women and elders, highlighting the necessity for focused interventions aimed at risk populations [29]. In another study, 38.5% of adults were classified as obese, with significant contributors identified as education level, dietary habits, and lack of physical activity [42]. Another study documented similar results in Dubai, indicating that 39.8% of adults were classified as overweight and obesity associated with various sociodemographic determinants such as age, sex, and occupation [43].

The influence of socioeconomic factors is substantial in the prevalence of obesity and various risk factors associated with NCDs. High educational levels correlated with physical activity that reduced obesity prevalence in Kuwait and Oman [33, 40]. In addition, obesity was prevalent, particularly among women, indicating that 47% of Omani women aged 30–49 years were classified as obese. Socio-demographic factors, such as marital status, income, and family structure, correlated with the behaviours related to meal timing and meal skipping in Kuwait [35]. These behaviours are consequently associated with an elevated risk of obesity and metabolic syndrome [33, 40].

The increasing incidence of childhood obesity presents a significant concern within the GCC region. Dietary consumption patterns and anthropometric measurements revealed that 28% of the children in the UAE were classified as overweight or obese. In addition, there were reported inadequacies in critical nutrients, including vitamins D and A [45]. Similarly, 42.3% of schoolchildren in Qatar were classified as overweight or obese, noting a greater prevalence among male students [44].

### Nutritional Patterns and Cardiovascular Risk Determinants

Some studies examine dietary patterns and their correlation with risk factors associated with cardiovascular disease (CVD). Kuwait showed three distinct dietary patterns, including the predominance of vegetables, the consumption of fast food, and a reliance on refined grains and poultry. Fast food consumption positively correlated with elevated BMI, increased waist circumference, and heightened blood pressure [32]. The consumption of refined grains and poultry was correlated with increased glucose levels. Similarly, inadequate dietary practices, characterised by a diminished consumption of nuts and seeds, excessive sodium intake, and insufficient fruit and vegetable intake, were responsible for 64.7% of cardiometabolic fatalities in Kuwait [34].

### Physical Activity and NCDs

Physical activity is a critical modifiable factor influencing high NCD risk in the GCC. Evidence from several studies shows that there is an inverse relationship between disease burden and physical activity levels. For instance [31], reported that, among elders, those with greater physical activity engagement presented with fewer NCDs, even after statistical adjustment for other variables. Population-based data also show that inactivity is widespread: in Saudi Arabia, almost 95% of adults were classified as insufficiently active [29, 33] while findings from Kuwait reveal that more active individuals were less likely to have obesity or central obesity [29, 33]. Physical inactivity in the region has been associated with high prevalence of NCDs such as hypertension, dyslipidemia, and type 2 diabetes [29, 33]. Barriers to regular activity include cultural norms, sedentary occupations, and environmental constraints such as high temperatures [30].

### Micronutrient Deficiencies and Malnutrition

Several studies examine the deficiencies of micronutrients and their implications for the risk of NCDs. About 23.8% of children in Oman were found to be anaemic, while 59.2% of women were classified as overweight or obese [39]. The prevalence of anaemia, iron deficiency, and micronutrient deficiency in Oman underscores the necessity for focused nutritional interventions [39]. Moreover, vitamin D deficiency was reported 67% among females in Al-Ain UAE [46]. Similarly, Bahrain was characterized by a low intake of fruits and vegetables alongside a high consumption of processed meats and sugary beverages. This dietary pattern is associated with nutritional deficiencies and a heightened prevalence of NCDs, including cardiovascular conditions [36].

### The Timing and Frequency of Meals

The correlation between meal timing and the incidence of NCDs has been examined across various nations within the GCC. There were distinct timing and frequency patterns in Kuwait, with 54% of Kuwaiti adults consuming food after 10 pm, while 29% skipped breakfast, noting a higher propensity for meal skipping among women in comparison to men [35]. The behaviours are associated with an elevated risk of obesity and may play a role in the development of additional metabolic disorders. Engaging in late-night eating and the practice of skipping meals have been recognized as notable behavioural risk factors for NCDs, especially in nations such as Kuwait and Bahrain, where the prevalence of unhealthy dietary habits was considerable [35]. During Ramadan, Muslims follow a unique eating schedule, with a mealtime shift to Suhoor and Iftar. The change in meal timing can significantly affect sleep patterns [50]. Better sleep quality is significantly related to adequate physical activity and adequate intake of vegetables, fruits, dates, and plant-based proteins. Conversely, consuming foods at night and smoking can disrupt sleep. This underscores how dietary choices during Ramadan can significantly affect overall health [50].

### Awareness and Educational Initiatives

Enhancing public health awareness concerning NCDs is essential for addressing the associated risk factors. About 70.6% of the Bahraini population possessed knowledge regarding Type 2 diabetes, with a notable increase in awareness among individuals diagnosed with the condition in contrast to those without diabetes [37]. The research advocated for educational initiatives to augment awareness and prevention strategies for Type 2 diabetes, highlighting the potential of social media as a tool for health communication [37]. In addition, enhanced education regarding nutrition, physical activity, and lifestyle modifications is critical to effectively manage obesity and improve diabetes outcomes [38, 43].

### Nutritional Quality of Life and Mental Health

In individuals with NCDs, there is an association between nutritional quality and mental health in the GCC. In Oman [38], reported a significant negative correlation between nutrition quality of life (NQOL) scores and symptoms of mental health, such as anxiety and depression, among individuals with type 2 diabetes, with 71.1% of participants indicating poor glycaemic control. The results show that (NQOL) influences metabolic outcomes and may also play a role in mental health prevalence. Related findings from other studies in the region support the interconnected nature of nutrition, physical health, and mental wellbeing [39]. Reported a high prevalence of overweight and obesity among Omani women (59.2%), while [40] found that socio-demographic factors such as income and marital status were significantly associated with obesity. These factors are recognised in the literature as potential influences on both quality of life and mental health. Together, these results underscore the need for integrated care approaches in NCD management that address nutrition, metabolic control, and psychological support simultaneously.

## Discussion

Our study summarized the prevalence of NCD in the region, representing a critical public health concern. The primary NCDs in the region, such as obesity, cardiovascular diseases, and type 2 diabetes, are closely linked to a combination of dietary, behavioural, and socioeconomic factors [52]. This study identified various risk factors contributing to the rising prevalence of NCDs in the GCC countries, including inadequate dietary habits, low physical activity levels, and socioeconomic inequalities.

Some studies observed an increase in the prevalence of obesity and overweight, particularly in Saudi Arabia, Kuwait, Oman, and the UAE. In Saudi Arabia, over 65% of the adult population is categorised as overweight or obese [29]. In Kuwait, data reveals that 42.1% of adults are classified as obese, with a significant 73.7% demonstrating central obesity [33]. In Qatar, over 42% of school-aged children are identified as overweight or obese, with the highest prevalence observed among Qatari male students [44]. The high prevalence of obesity reflects a complex interplay of dietary practices, insufficient physical activity, and socio-demographic factors, such as lower education and income levels, which are commonly recognized as key correlates of obesity in these countries [30, 42].

The dietary patterns associated with obesity and various NCDs in the GCC region are marked by a high intake of fast foods, refined grains, and sugary beverages, alongside a reduced consumption of fruits and vegetables [14, 15]. Research in Kuwait highlighted the adverse effects of a fast-food diet on BMI and waist circumference and its association with elevated blood pressure [32]. Similar patterns were identified in Bahrain, where inadequate consumption of fruits and vegetables, high intake of processed meats and sugary beverages were considered critical dietary risk factors for NCDs [36]. In addition, insufficient physical activity constitutes a significant risk factor for NCDs in the region [53].

Socioeconomic factors significantly influenced the prevalence of NCDs [51, 54]. Individuals from lower socioeconomic backgrounds, especially those with reduced educational attainment and income, demonstrate a higher prevalence of NCDs [29]. In Bahrain, the prevalence of NCDs was notably higher among individuals with lower educational attainment and income levels [30]. The identified disparities highlight the need for targeted public health interventions emphasizing socioeconomic equity. In Oman, several socio-demographic characteristics, such as marital status, income, and family structure, significantly correlated with obesity, particularly among women [40]. Similarly, lower educational attainment, inadequate dietary practices, and insufficient physical activity were associated with obesity in the United Arab Emirates [42]. These findings suggest that strategies to improve academic outcomes, promote healthy nutritional habits, and encourage physical activity could help reduce the rising prevalence of non-communicable diseases in these countries.

This study emphasizes the importance of national policy interventions in tackling nutrition-related NCDs. In addition, inadequate nutrition linked to cardiometabolic mortality in Kuwait highlights a need for nutritional interventions targeting high-risk groups, especially young adults, and males [34]. The implementation of an extensive framework for dietary guidelines in Bahrain, addressing physical health, mental wellness, cultural heritage, and environmental factors, demonstrates the importance of integrating multiple aspects of health into public health policies aimed at reducing the effects of NCDs [36].

These results highlight the critical influence of lifestyle behaviours, including physical activity, nutrition, and stress management, on long-term health outcomes. The significant prevalence of Type 2 diabetes in GCC countries emphasises the detrimental effects of unhealthy lifestyles, such as inadequate diet and lack of physical activity [16]. The increasing prevalence of diabetes in the GCC, influenced by obesity and genetic factors, reflects the issues identified related to inadequate dietary practices and insufficient physical activity among adolescents, which heighten the risk of future chronic diseases. The findings underscore the necessity for early interventions aimed at modifiable risk factors within the youth, especially in areas such as the GCC, where lifestyle-related diseases are increasing [35].

A conceptual framework linking dietary and lifestyle factors to NCDs in GCC countries can be understood through the region’s rapid nutrition and lifestyle transition. Economic growth, urbanization, and globalization have increased the availability of energy-dense processed foods, sugary beverages, and fast foods, while reducing adherence to traditional diets rich in whole grains, fruits, and vegetables. Concurrently, sedentary lifestyles, driven by car-dependent environments, technology use, and extreme climatic conditions limiting outdoor physical activity, contribute to obesity and metabolic disorders. These behavioural and environmental factors interact with socioeconomic determinants and genetic susceptibility to increase the risk of obesity, type 2 diabetes, and cardiovascular diseases in GCC populations.

Beyond the observed prevalence and associations, the rising burden of NCDs in GCC countries reflects broader structural and environmental transitions that influence health behaviours. Rapid economic development, urbanization, and modernization in the region have contributed to a nutrition transition, characterized by increased availability of energy-dense processed foods and reduced reliance on traditional diets rich in whole grains, fruits, and legumes. At the same time, built environment factors, including car-dependent urban design, extreme climatic conditions limiting outdoor activity, and sedentary occupational patterns, reduce opportunities for physical activity. Cultural and social norms, including dietary practices, hospitality-related food consumption, and gender-related barriers to physical activity in some settings, may further shape behavioural risk factors. These contextual influences interact with genetic susceptibility to obesity and insulin resistance reported in Middle Eastern populations, creating a complex causal pathway linking lifestyle changes to the high prevalence of obesity, type 2 diabetes, and cardiovascular diseases in the GCC. Understanding these mechanisms is essential for designing culturally appropriate and context-specific public health interventions.

### Study Strengths and Limitations

The present review included recent studies published between 2020 and 2024, providing up-to-date evidence on nutrition-related risk factors, including unhealthy dietary patterns, socioeconomic determinants, lack of physical activity, and behavioural risk factors, influencing the increasing prevalence of NCDs. This date range is a strength, as it synthesizes the most recent evidence reflecting the current GCC policy context and the post-2020 period, including pandemic-related disruptions to diet and physical activity. In addition, the countries within the GCC present comparable fast rates of economic development, urbanization, and shared public health problems and nutritional behaviours. This enables the applicability of the findings within the broader GCC and other population groups, including adults, children, and migrants, to inform the targeting of public health strategies.

However, most included studies employed cross-sectional designs, which preclude the establishment of causal relationships between nutritional risk factors and NCDs. The absence of longitudinal data limits understanding of risk factor exposure and disease development temporal sequence. In addition, many studies utilized self-reported questionnaires for data collection, particularly regarding dietary habits and physical activity levels. These may introduce recall and social desirability biases, potentially leading to underreporting unhealthy behaviors and overreporting healthy practices. Moreover, the variability in the instruments used to assess dietary patterns, physical activity, and anthropometric measurements across studies limits direct comparability and may have influenced prevalence estimates of risk factors.

### Implications and Recommendations

Future research should evaluate the effectiveness of specific intervention programs aimed at reducing nutrition- and lifestyle-related risk factors for NCDs in GCC countries. For example, school-based nutrition and physical activity programs targeting adolescents and adults could improve dietary habits, increase physical activity levels, and reduce overweight and obesity prevalence. Similarly, community-based lifestyle modification programs for adults such as structured weight-management initiatives, dietary counselling, and physical activity promotion may help reduce cardiometabolic risk factors including obesity, hypertension, and type 2 diabetes.

At the policy level, regulatory authorities in GCC countries could implement population-level strategies, such as taxation on sugar-sweetened beverages, front-of-package nutrition labeling, and restrictions on marketing unhealthy foods. These measures may help reduce the consumption of ultra-processed foods and sugary beverages while encouraging healthier dietary choices. Additionally, workplace wellness programs promoting regular physical activity, healthy food environments, and routine health screenings could contribute to early detection and prevention of NCDs among working-age adults.

Healthcare systems should also strengthen preventive services, including routine screening for obesity, hypertension, and diabetes in primary healthcare settings, coupled with lifestyle counselling and referral to nutrition or lifestyle medicine programs. Integrating multisectoral strategies involving health, education, and urban planning sectors such as improving access to recreational spaces and promoting active transport could further support healthier lifestyles.

Future studies should also employ longitudinal designs to assess the long-term impact of lifestyle interventions on NCD prevention in GCC populations, while exploring how socioeconomic, cultural, and environmental factors influence dietary behaviours and health outcomes. Such evidence would provide policymakers with clearer guidance for implementing targeted and effective public health interventions.

### Conclusion

This review provides evidence of the substaintial burden of lifestyle-related NCDs across the GCC region. Dietary practices, physical inactivity, and socioeconomic factors influence the prevalence of obesity, cardiovascular diseases, and type 2 diabetes in these countries. In addition, the nutritional landscape of the GCC region is characterized by concerning dietary patterns that include excessive consumption of fast foods, refined grains, and sugar-sweetened beverages, coupled with inadequate intake of fruits, vegetables, and whole grains. These patterns, alongside widespread physical inactivity, promote the development and progression of NCDs. Furthermore, the identified socioeconomic gradients in NCDs prevalence, with higher burdens among less educated and lower-income groups, especially women, underscore the need for equity-focused approaches to health promotion. The high prevalence of childhood obesity across the region highlights an impending crisis that will likely translate into even higher adult NCD rates in the coming decades. Early intervention programs focusing on school-based nutrition education, mandatory physical education, and creating supportive environments for healthy behaviors are critical to addressing the issue. Public health policy should focus on nutrition, health improvement, physical activity promotion, and socioeconomic inequality. Strengthening public education, dietary guideline adherence, and physical exercise promotion to minimize NCDs in the GCC is also essential.
